# MLN-4760 Induces Oxidative Stress without Blood Pressure and Behavioural Alterations in SHRs: Roles of *Nfe2l2* Gene, Nitric Oxide and Hydrogen Sulfide

**DOI:** 10.3390/antiox11122385

**Published:** 2022-12-01

**Authors:** Michal Kluknavsky, Andrea Micurova, Martina Cebova, Ezgi Şaman, Sona Cacanyiova, Iveta Bernatova

**Affiliations:** Centre of Experimental Medicine, Slovak Academy of Sciences, Institute of Normal and Pathological Physiology, 841 04 Bratislava, Slovakia

**Keywords:** ACE2, antioxidant enzymes, oxidative damage, nitric oxide, hydrogen sulfide, NRF2, inflammation

## Abstract

Reduced angiotensin 1–7 bioavailability due to inhibition of angiotensin-converting enzyme 2 (ACE2) may contribute to increased mortality in hypertensive individuals during COVID-19. However, effects of ACE2 inhibitor MLN-4760 in brain functions remain unknown. We investigated the selected behavioural and hemodynamic parameters in spontaneously hypertensive rats (SHRs) after a 2-week s.c. infusion of MLN-4760 (dose 1 mg/kg/day). The biochemical and molecular effects of MLN-4760 were investigated in the brainstem and blood plasma. MLN-4760 had no effects on hemodynamic and behavioural parameters. However, MLN-4760 increased plasma hydrogen sulfide (H_2_S) level and total nitric oxide (NO) synthase activity and conjugated dienes in the brainstem. Increased NO synthase activity correlated positively with gene expression of *Nos3* while plasma H_2_S levels correlated positively with gene expressions of H_2_S-producing enzymes *Mpst*, *Cth* and *Cbs*. MLN-4760 administration increased gene expression of *Ace2*, *Sod1*, *Sod2*, *Gpx4* and *Hmox1*, which positively correlated with expression of *Nfe2l2* gene encoding the redox-sensitive transcription factor NRF2. Collectively, MLN-4760 did not exacerbate pre-existing hypertension and behavioural hyperactivity/anxiety in SHRs. However, MLN-4760-induced oxidative damage in brainstem was associated with activation of NO- and H_2_S-mediated compensatory mechanisms and with increased gene expression of antioxidant, NO- and H_2_S-producing enzymes that all correlated positively with elevated *Nfe2l2* expression.

## 1. Introduction

The renin–angiotensin system (RAS) is a major regulator of cardiovascular function, playing a pivotal role in the blood pressure (BP) regulation. The classical RAS consists of circulating renin, acting on angiotensinogen to produce angiotensin I (Ang I), which in turn is converted to angiotensin II (Ang II) by angiotensin-converting enzyme (ACE). Ang II was considered a major effector of RAS and its physiological effects dependent on the binding to the angiotensin receptors (ATR1 or ATR2). In addition, several studies confirmed the significant regulatory role of angiotensin-(1-7) (Ang-(1-7)), which is generated predominantly by angiotensin-converting enzyme 2 (ACE2) [1]. ACE2 is also considered an important therapeutic target in controlling the coronavirus disease-19 (COVID-19) outbreak, since severe acute respiratory syndrome coronavirus 2 (SARS-CoV-2) uses ACE2 as the receptor to enter the cells [2,3].

There are studies that suggest ACE2 as a suitable target in treatment of COVID-19 [4,5,6]. On the other hand, SARS-CoV-2 binding to ACE2 may lead to reduced Ang-(1-7) bioavailability, which may contribute to deteriorating Mas receptor (MasR, proto-oncogene and G protein-coupled receptor) signal transduction mediated by nitric oxide (NO) [7,8]. Thus reduced ACE2/MasR/NO-mediated signaling may contribute to worsened vascular and cardiac functions in patients with COVID-19, especially in those with pre-existing hypertension. In our study, we used spontaneously hypertensive rats (SHRs) as a model of human essential hypertension. Various studies characterized features of SHRs such as increased systolic BP, cardiovascular hypertrophy, age-dependent endothelial dysfunction and increased systemic resistance [9,10,11]. In addition, SHRs show pulmonary complications such as inflammation and oxidative stress [12,13]. Thus, increased lung sensitivity in SHRs may represent a potential model of pulmonary injury, similar to that in patients with COVID-19 suffering from hypertension. In addition, SHRs also serve as an experimental model of attention deficit hyperactivity disorder (ADHD) due to their locomotor hyperactivity and reduced anxiety [14]. Regarding the role of ACE2/Ang-(1-7)/MasR pathway in modulation of behaviour, several studies reported reduced anxiety-like behaviour in rodents after administration of an ACE2 activator and Ang-(1-7), respectively, or induced by overexpression of ACE2 [15,16,17,18]. However, to the best of our knowledge, the effects of the ACE2 inhibitor MLN-4760 on brain function and behaviour are unknown in either SHRs or other rodent models.

NO is an important vasodilator in the CVS that balances the effects of vasoconstrictors [19]. Several authors have found increased NO production after Ang-(1-7) administration. Binding of Ang-(1-7) to the MasR led to activation of the endothelial isoform of NO synthase (NOS) [20,21]. In addition, multiple studies described NO as a potent neurotransmitter and/or neuromodulator in the central and peripheral nervous systems [22,23]. Therefore, alterations in NO production affect both cardiovascular functions and behaviour. Indeed, neuronal NOS was shown to be involved in various behavioural abnormalities, including ADHD [24,25].

Another important gasotransmitter is the H_2_S. Although H_2_S is produced by neuronal cells in various brain regions, a study by Lee et al. revealed that the main source of H_2_S production are glial cells, specifically astrocytes [26,27]. The disturbances in H_2_S levels and trans-sulfuration pathway have been implicated in neurodegenerative disorders such as Alzheimer’s disease, Parkinson disease, stroke and traumatic brain injury. In addition, H_2_S is considered as a potent antioxidant, anti-inflammatory and anti-apoptotic molecule suggesting its neuroprotective potential [28]. Recent studies showed that intra- and intermolecular disulfides in ACE2 molecule had an important role in SARS-CoV-2 binding to ACE2. When the disulfides of ACE2 were reduced to sulfhydryl groups, the virus binding became weaker [29,30].

The nuclear factor erythroid 2 p45-related factor 2 (NRF2) is a master regulator of multiple cytoprotective responses restoring redox and protein homeostasis, promoting resolution of inflammation and facilitating repair [31]. Its role in various non-communicable diseases was reviewed previously [32]. As oxidative stress and low-grade inflammation are present in hypertension, NRF2-mediated antioxidant and anti-inflammatory mechanisms, together with ligand-dependent nuclear receptor peroxisome proliferator-activated receptor gamma activation, play a significant role in prevention and treatment of cardiovascular and metabolic diseases, including hypertension [32]. In addition, due to its anti-inflammatory action, the activation of NFR2 has been suggested as a possible strategy against COVID-19 [33].

The aim of our study was to examine the effects of long-term administration of the specific inhibitor ACE2 MLN-4760 on open field and elevated plus maze behaviour in SHRs. The study focused on determining H_2_S formation in plasma and on NO and oxidative changes in the brainstem (BS). In addition, we investigated expression of genes involved in NO and H_2_S production as well as in antioxidant defence in the BS. We tested the hypothesis that MLN-4760 alters neuronal NO and H_2_S signaling in the BS region, which is significantly involved in the BP regulation and the integration of the behavioural response. We hypothesized that administration of ACE2 inhibitor MLN-4760 may lead to exacerbation of pre-existing hypertension and/or behavioural abnormalities in SHRs in association with oxidative stress and inflammation.

## 2. Materials and Methods

### 2.1. Animals

The SHRs used in our study were purchased from the accredited breeding facility Dobra Voda, which falls under the Centre of Experimental Medicine, Slovak Academy of Sciences, Slovak Republic. Rats were bred in accordance with the institutional guidelines of the Ethical Committee of the Centre of Experimental Medicine. The animals were housed in a 12 h light/12 h dark cycle at constant humidity (45–65%) and temperature (20–22 °C) and had free access to standard laboratory rat chow (Altromin 1324P, Altromin International, Lage, Germany) and drinking water ad libitum.

### 2.2. Experimental Design

We used 16- to 18-week-old male SHRs (*n* = 22) in our study. All rats were divided into control group (Cont; *n* = 11) and group treated with MLN-4760 (MLN; *n* = 11). The specific ACE2 inhibitor MLN-4760 (MedChemExpress, Monmouth Junction, NJ, USA) was administered by mini-osmotic pumps (Alzet^®^, model 2002, Durect^TM^, Cupertino, CA, USA) with a pumping rate of 0.5 µL/h. An amount of MLN-4760 corresponding to a daily dose of 1 mg/kg/day of MLN-4760 was dissolved in 10% dimethyl sulfoxide in isotonic saline solution (NaCl 0.9% intravenous solution for infusion; B. Braun Melsungen AG, Melsungen, Germany) and continuously infused s.c. for 14 days. Day of minipump implantation counts as day 0 of treatment. In the control group, mini-osmotic pumps were filled with 10% dimethyl sulfoxide in isotonic saline. A detailed description of the surgical procedures is provided in the previous study [34]. After the 14 days of treatment, the rats were killed by decapitation after brief CO_2_ anaesthesia. After decapitation, trunk blood was collected into heparinized tubes (140 UI/5 mL) and aliquots were stored at −80 °C. BS samples were rapidly dissected, weighed, frozen in liquid nitrogen, and stored at −80 °C until further analyses.

### 2.3. Systolic BP and Heart Rate Determination

The systolic BP and heart rate (HR) was measured in preconditioned, conscious rats by using non-invasive tail-cuff plethysmography (MRBP, IITC Life Science Inc., Los Angeles, CA, USA) between 08:00 a.m. and 12:00 a.m. Systolic BP and HR were determined in the control group (*n* = 11) and MLN group (*n* = 11). All rats were trained for the tail-cuff method of BP measurement for 3 consecutive days before determination of basal levels of BP. Basal systolic BP was measured 3 days before minipump implantation. Final systolic BP (end) was determined on the 13th day of treatment. BP of all rats was measured five times and BP was calculated as the average of the last four measurements.

### 2.4. Testing Exploratory Behaviour and Anxiety-Like Behaviour

Exploration (i.e., spontaneous locomotion) and anxiety-like behaviour were investigated using the open-field test (OF) and elevated plus maze test (EPM) between 08:00 a.m. and 12:00 a.m. Behavioural testing by OF and EPM was determined in the control group (*n* = 11) and MLN group (*n* = 11). Rats, in their home cages, were placed into a test room with the lighting and environmental conditions described above, approximately 12 h before the test. OF test was performed two times, 2 days before basal BP measurements (basal) and on the 12th day of treatment (end) to avoid the effect of behavioural testing on BP levels. EPM test was performed only on the 12th day of treatment, approximately 1 h after the OF test. Rats were kept in their home cage, in the testing room, between the tests. Testing conditions were described in detail previously [35].

At the beginning of behavioural tests, individual rats were placed in the centre of the OF and EPM, respectively. Locomotor activity was recorded and evaluated by ANY-maze video-tracking software (Stoelting, Wood Dale, IL, USA) for 10 min in the OF or for 5 min in the EPM. The OF and EPM area were cleaned with soapy water and dried with paper towels after each rat. The following behavioural parameters were determined in both OF and EPM: total distance travelled and time of immobility as the parameters of exploratory behaviour. Anxiety-like behaviour parameters in the OF test included distance travelled and time spent in the central zone. The time spent in the open and closed arms as well as the number of entries into the open and closed arms were used as parameters of anxiety-like behaviour in the EPM test. By repeated testing in the OF, the degree of habituation of exploratory behaviour and anxiety-like behaviour in the OF were investigated.

### 2.5. Total NO Synthase Activity

Total NOS activity was determined in the 10% of BS homogenates by measuring [3H]-L-citrulline formation from [3H]-L-arginine (MP Biochemicals, Santa Ana, CA, USA) using the Quanta Smart Tri-Carb Liquid Scintillation Analyzer (PerkinElmer, Beaconsfield, UK) as described in previous work [36]. Total NO synthase activity was determined in the control group (*n* = 7) and MLN group (*n* = 7). The results are expressed as picokatal per gram of protein (pkat/g protein).

### 2.6. Measurement of Plasma H_2_S Concentration

H_2_S concentration was measured in plasma via methylene blue assay as described previously [37]. Plasma H_2_S concentration was determined in the control group (*n* = 7) and MLN group (*n* = 7). To determine H_2_S formation, 75 µL of plasma was combined with 500 µL of reaction mixture containing 0.1 mol/L potassium phosphate buffer (325 µL), substrate and H_2_S cofactors that are L-cysteine and pyridoxal-5-phosphate, respectively. Samples were incubated for 30 min at 37 °C. After the incubation, 10% trichloroacetic acid (250 mL) was added to the mixture followed by 1% zinc acetate (250 mL), N,N-dimethyl-p-phenylenediamine sulfate (20 mmol/L, 133 mL) in 7.2 mol/L HCl, and FeCl_3_ (30 mmol/L, 133 mL) in 1.2 mol/L HCl incubation to trap H_2_S and to precipitate proteins. After 10 min of incubation at room temperature, the mixtures were centrifuged for 5 min, 8944× *g* at 4 °C. In a 96-well plate, all standards and samples were assayed in duplicated. The H_2_S concentration of each sample was calculated against a calibration curve of (Na2S; 3.9–250 mmol/L) and results show the plasma H_2_S concentration in µmol/L. The absorbance of resulting solution was measured at 650 nm with a spectrophotometer (NanoDrop™ 2000/2000c Spectrophotometers, Thermo Fisher Scientific, Waltham, MA, USA). Protein concentration was determined by Lowry assay.

### 2.7. Measurement of Conjugated Dienes Content

Conjugated dienes (CD), as a marker of lipid peroxidation and oxidative damage, were measured in 10% tissue homogenates of the BS. The exact methodological procedure for the processing and isolation of CD in tissue samples was described in detail previously [38]. CD content was determined in the control group (*n* = 7) and MLN group (*n* = 7). The absorbance of the samples was measured at 233 nm. An extinction coefficient of 26,000 mol^–1^·L·cm^–1^ was used for calculation of results. The results were expressed in nanomole of CD per gram of tissue (nmol/g).

### 2.8. Gene Expression Determination

The gene expression levels of neuronal NOS (*Nos1*), inducible NOS (*Nos2*), endothelial NOS (*Nos3*), nuclear factor erythroid 2 p45-related factor 2 (*Nfe2l2*), peroxisome proliferator activated receptor gamma (*Pparg*), superoxide dismutase 1 (*Sod1*) and superoxide dismutase 2 (*Sod2*), glutathione peroxidase 4 (*Gpx4*), heme oxygenase 1 (*Hmox1*), 3-mercaptopyruvate sulfurtransferase (*Mpst*), cystathionine gamma-lyase (*Cth*), cystathionine-β-synthase (*Cbs*), tumor necrosis factor alpha (*Tnf*), interleukin 1 beta (*Il1b*), cyclooxygenase 1 and cyclooxygenase 2 (*Ptgs1* and *Ptgs2*), angiotensin-converting enzyme 2 (*Ace2*), Mas receptor (*Mas1*) and 60S ribosomal protein L10a (*Rpl10a*, housekeeping gene) in the BS tissue were determined by using real time quantitative polymerase chain reaction (qPCR).

The total RNA was isolated using the PureZOL™ RNA Isolation Reagent (Bio-Rad, Hercules, CA, USA), according to the manufacturer’s protocols. The amount and purity of total isolated RNA was spectrophotometrically quantified at 260/280 nm and 260/230 nm while using a NanoDrop spectrophotometer (Thermo Scientific, Waltham, MA, USA). Reverse transcription was performed using 1 μg of total RNA from each sample using Eppendorf Mastercycler (Eppendorf, Hamburg, Germany) and an iScript-Reverse Transcription Supermix (Bio-Rad, Hercules, CA, USA), according to the manufacturer’s protocols. Gene-specific primers were designed using the PubMed program (Primer-BLAST) and database (Gene). The DNA sequences and melting temperature of the used primers, the size of the amplicons and the reference numbers of the templates are described in Table 1.

The PCR reactions were conducted in a final volume of 20 μL containing 2 μL of 5-fold diluted template cDNA, 10 μL SsoAdvanced mix (SsoAdvanced Universal SYBR Green Supermix, Bio-Rad, Hercules, CA, USA), 1.5 μL of both forward and reverse primers (Metabion, Planegg, Germany, 4 μmol/L), and 5 μL RNAase free water (Merck, Darmstadt, Germany (previously Sigma–Aldrich)) in a final volume of 20 μL. The thermal cycling conditions were as follows: (1) 95 °C for 30 s, (2) 40 cycles consisting of (a) 95 °C for 10 s, (b) an optimal annealing temperature (depending on the selected primer, see Table 1) for 20 s. Finally, melt curves for amplicon analyses were constructed at 60–95 °C, 5 s/1 °C. PCR method was performed using a CFX96 Real-Time PCR (Bio–Rad, Hercules, CA, USA) detection system and evaluated by Bio–Rad CFX Manager software 2.0 (Bio–Rad, Hercules, CA, USA). Expression of each gene was determined in Cont group (*n* = 7) and MLN group (*n* = 7). After completion of reaction, data analysis has been performed. For each sample, the Ct values of target genes and Ct values of housekeeping gene *Rpl10a* were used to estimate relative change in specific gene expression. Relative expressions of genes were calculated using the 2^−ΔΔCT^ method [39].

### 2.9. Statistical Analysis

The normality of the data was analysed by Shapiro–Wilk W test. Results were analysed by repeated measures ANOVA (measurements, i.e., basal and end as repeated factor) or Student’s t-test were appropriate. ANOVA analyses were followed with Bonferroni’s post-hoc test. The results are presented as mean ± standard error of means (SEM). Correlations between variables were evaluated using Pearson’s correlation coefficient (r). The differences between the means were assessed as significant at *p* < 0.05. GraphPad Prism v7.02 (GraphPad Software, Inc., San Diego, CA, USA) and Statistica v13.5 (StatSoft Europe, Hamburg, Germany) were used for the statistical analyses.

## 3. Results

### 3.1. Hemodynamic Parameters of Experimental Animals

No significant changes were found in end values of systolic BP and HR in both the control and MLN group compared to their basal values. There were also no differences in the end values of systolic BP and HR between the control and MLN group. The exact values of all parameters are presented in Table 2.

### 3.2. Behavioural Analysis of Exploration and Anxiety-Like Behaviour

**Figure 1:**
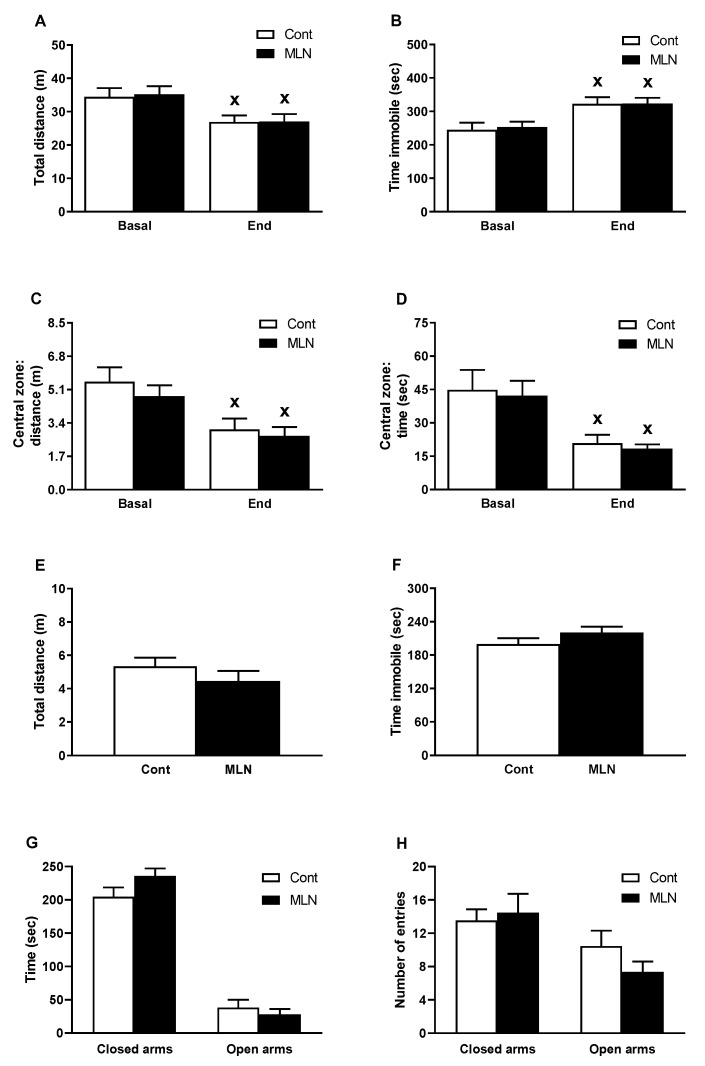
Effect of MLN-4760 on exploratory behaviour and anxiety-like behaviour in OF (**A**–**D**) and EPM (**E**–**H**) test. Values represent the mean ± SEM (*n* = 11 per group). ^x^ *p* < 0.05 vs. basal values of the same group. Data were analyzed by repeated measures ANOVA (OF test) or Student’s *t*-test (EPM test). ANOVA analysis was followed with Bonferroni’s post-hoc test. Abbreviations: Cont, control group; MLN, MLN-4760-treated group.

In the OF, administration of MLN-4760 had no effect on end values of total distance travelled (Figure 1A), total immobility time (Figure 1B), distance travelled (Figure 1C) and time spent (Figure 1D) in the central zone compared to the control group. In the MLN group, repeat testing in OF revealed the similar degree of habituation in parameters of exploratory behaviour (Figure 1A,B) and parameters of anxiety-like behaviour (Figure 1C,D) as it was found in the control group. In the EPM, administration of MLN-4760 did not alter total distance travelled (Figure 1E), time of immobility (Figure 1F), time spent (Figure 1G) and number of entries (Figure 1H) in the open and closed arms (parameters of anxiety-like behaviour) compared to the control group.

### 3.3. Total NOS Activity and Expression of Nos3-1, Ace2 and Mas1 Genes

**Figure 2:**
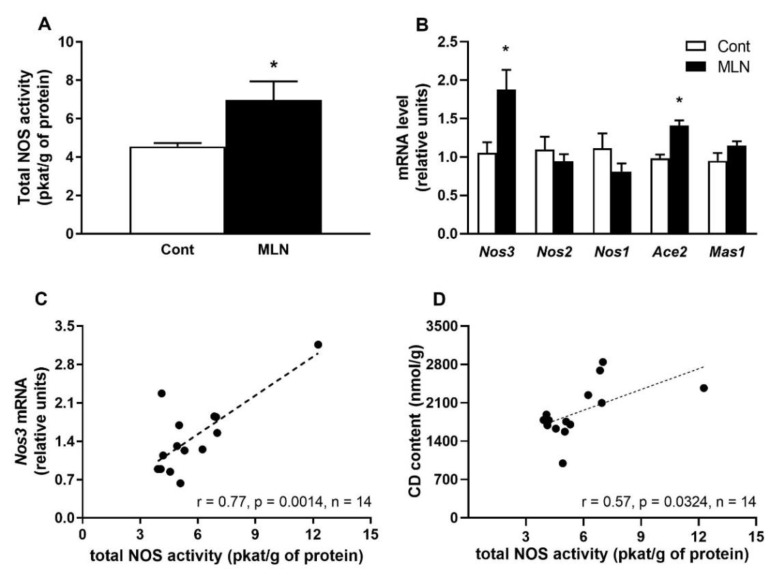
Effect of MLN-4760 on total NOS activity (**A**), gene expressions of NO-producing enzymes (*Nos3*-*1*) and genes encoding proteins involved in ACE2 pathway (*Ace2*, *Mas1*) (**B**). Total NOS activity correlations with *Nos3* gene expression (**C**) and with the level of conjugated dienes (CD) level (**D**). Values represent the mean ± SEM (*n* = 7 per group). * *p* < 0.05 vs. control group. Data were analysed by Student’s *t*-test.

Total NOS activity was significantly (*p* = 0.03) elevated in MLN group compared to the control group (Figure 2A). Administration of MLN-4760 also led to a significant increased expression in *Nos3* (*p* = 0.014) and *Ace2* (*p* = 0.0002) genes compared to the control group (Figure 2B). The expressions of *Nos2*, *Nos1* and *Mas1* genes were not affected by MLN-4760 treatment (Figure 2B). Further statistical analysis revealed that total NOS activity positively correlated with gene expression of *Nos3* gene (Figure 2C) as well as with CD content (Figure 2D).

### 3.4. Oxidative Damage and Expression of Genes Involved in Antioxidant Defence and Inflammatory Responses

**Figure 3:**
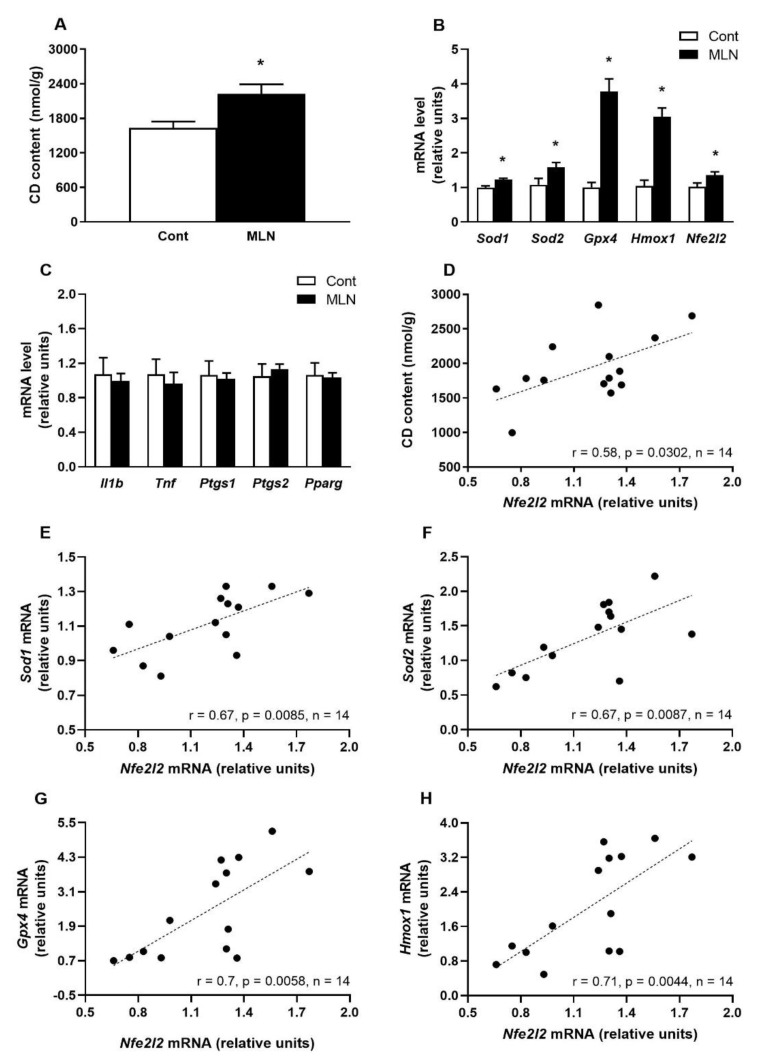
Effect of MLN-4760 on CD content (**A**) as well as expressions of genes encoding proteins involved in antioxidant defence (**B**) and inflammatory response (**C**). *Nfe2l2* gene expression correlations with CD content (**D**) and with gene expression of antioxidants (**E**–**H**). Values represent the mean ± SEM (*n* = 7 per group). * *p* < 0.05 vs. the control group. Data were analysed by Student’s *t*-test.

MLN-4760 administration significantly (*p* = 0.032) elevated CD content in the BS compared to the control group (Figure 3A). The increased CD content was accompanied by a significantly increased gene expression of antioxidant enzymes *Sod1* (*p* = 0.006) *Sod2* (*p* = 0.048), *Gpx4* (*p* = 0.0001), *Hmox1* (*p* = 0.0003) and redox sensitive transcription factor *Nfe2l2* (*p* = 0.041) compared to the control group (Figure 3B). Between the control and MLN group, there were no differences in the expression of *Il1b*, *Tnf*, *Ptgs1*, *Ptgs2* genes associated with the inflammatory pathway and the gene expression of the anti-inflammatory transcription factor *Pparg* (Figure 3C). Further statistical analysis revealed that expression of *Nfe2l2* gene positively correlated with CD content associated with oxidative damage (Figure 3D) as well as with expression of *Sod1*, *Sod2*, *Gpx4* and *Hmox1* genes encoding antioxidant enzymes (Figure 3E–H).

### 3.5. Plasma Level of H_2_S and Gene Expression H_2_S-Producing Enzymes

**Figure 4:**
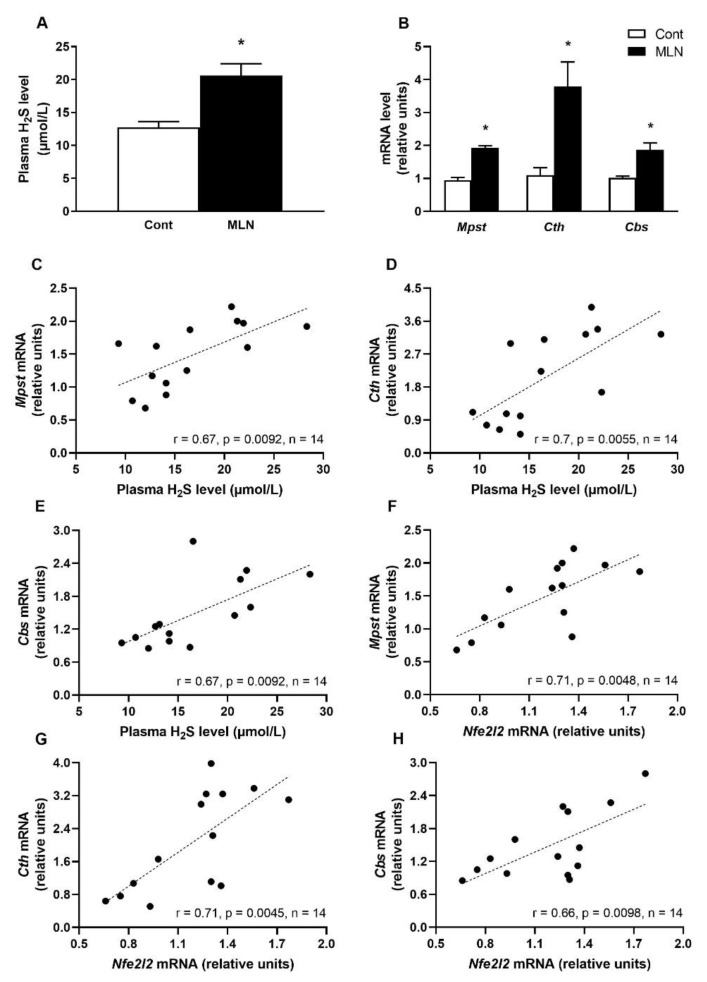
Effect of MLN-4760 on plasma H_2_S levels (**A**) and gene expressions of H_2_S-producing enzymes (**B**). Plasma H_2_S level correlations with expression of *Mpst*, *Cth* and *Cbs* genes (**C**–**E**). *Nfe2l2* gene correlations with the expression of *Mpst*, *Cth* and *Cbs* genes (**F**–**H**). Values represent the mean ± SEM (*n* = 7 per group). * *p* < 0.05 vs. the control group. Data were analysed by Student’s *t*-test.

Plasma H_2_S level was significantly (*p* = 0.002) increased after MLN-4760 treatment compared to the control group (Figure 4A). The increased plasma H_2_S level was accompanied by a significantly increased gene expression of *Mpst* (*p* = 0.0001), *Cth* (*p* = 0.0001) and *Cbs* (*p* = 0.0007) in the MLN group compared to the control group (Figure 4B). Further statistical analysis revealed that expressions of *Mpst*, *Cth* and *Cbs* genes positively correlated with plasma H_2_S level as well as with expression of *Nfe2l2* gene (Figure 4C–H).

## 4. Discussion

Several human studies reported an increased risk for anxiety and mood disorders during and after COVID-19 disease while cardiovascular and metabolic diseases represent significant risk factors for development of serious complications during and/or post COVID-19 [40,41]. However, little is known whether reduction of the Ang-(1-7) formation, which has been shown to induce anxiolytic and BP reducing effects, can worsen existing hypertension and/or cause behavioural disorders in SHRs. We focused on the effects of MLN-4760 on systolic BP, behaviour, changes in oxidation state, NO and H_2_S production, and corresponding changes in expression of genes involved in antioxidant defence, pro-inflammatory response, NO- and H_2_S-producing enzymes, as well as transcription factor NRF2 in BS of SHR rats.

Several studies have reported behavioural effects associated with the ACE2/ Ang-(1-7)/MasR signaling pathway. Wang et al. reported anxiolytic effects due to overexpression of ACE2, as well as after i.c.v. administration of an ACE2 agonist or a MasR antagonist, respectively [17]. Similar anxiolytic effects were also in found after i.c.v. administration of Ang-(1–7) which reversed the anxiety-like behaviour in transgenic (mRen2)27 hypertensive rats [42,43]. Intragastric administration of ACE inhibitor perindopril or angiotensin receptor blocker candesartan led to increased MasR protein expression, ACE2 activity and Ang-(1-7) level in the hippocampus, which was associated with increased anxiolytic effects in SHRs with chronic cerebral hypoperfusion [44]. Based on the abovementioned studies, the activation/inhibition of the ACE2/ Ang-(1-7)/MasR pathway produces behavioural alterations manifested by overall improvement/worsening of anxiety-like behaviour. We found that chronic low-dose administration of MLN-4760, which elevated *Ace2* gene expression, had no effects on exploratory behaviour, anxiety-like behaviour and rate of habituation in SHRs.

The altered expression of ACE2 enzyme can also play an important role in the development of primary hypertension thus enhancing ACE2 activity/expression may be a useful therapeutic approach in the management of high BP. In several studies, administration of ACE2 agonists led to increased ACE2 activity associated with reduced BP in SHRs [45,46]. Similar effects were also described regarding overexpression of the ACE2 enzyme in SHRs. Díez-Freire et al. found that overexpression of ACE2 in the heart of 5-day-old SHRs attenuated the development of hypertension in ~17-week-old [47]. Overexpression of ACE2 in rostral ventrolateral medulla of SHRs also caused a significant BP reduction [48]. In different rodent model, ACE2 overexpression in the heart and hypothalamic paraventricular nucleus led to a decrease in BP whose increase was induced by Ang II infusion in Sprague–Dawley rats [49,50]. Based on the abovementioned studies, one might assume that inhibition ACE2 will lead to BP rising. However, study of Diz et al. showed reduced BP up to 90 min after injection of MLN-4760 into nucleus tractus solitarii of normotensive Sprague–Dawley rats [51]. In our studies [34], 14-day s.c. infusion of MLN-4760 failed to alter systolic BP and HR in SHRs, similarly as it was found in this study. The absence of behaviour and BP changes after chronic MLN-4760 treatment may result from the increased expression of the *Ace2* gene due to the activation of compensatory mechanisms in order to maintain normal ACE2 signalling in the BS. These might be associated with apelin, sirtuin 1 or adenosine monophosphate kinase-mediated transcriptional regulation of ACE2 [52].

**Figure 5:**
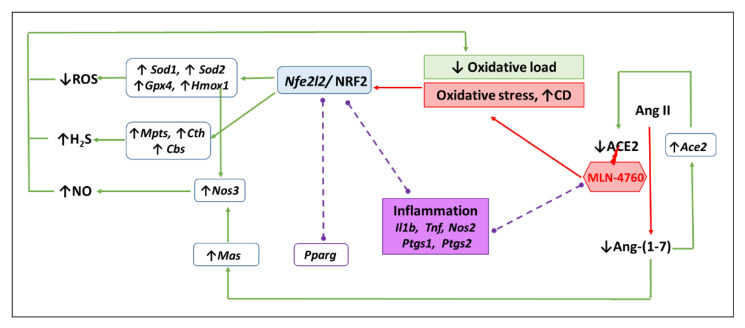
Scheme of a putative effect of MLN-4760 on the expression of antioxidant and inflammatory genes. MLN-4760 can, at least temporarily, reduce the level of Ang-(1-7), which can, on the principle of negative feedback, lead to increased expression of the *Ace2* gene in order to restore the production of Ang-(1-7) by the ACE2. A low level of Ang-(1-7) can lead to increased gene expression of *Mas* (encoding Mas receptor) in order to increase signaling through this receptor. We assume that MLN-4760 can induce oxidative stress determined by elevated levels of conjugated diene (CD), subsequently increase *Nfe2l2* expression and activate NRF2 target antioxidant genes (*Sod1*, *Sod2*, *Gpx4*, *Hmox1*). Decreased production of reactive oxygen species, increased production of nitric oxide associated with elevated *Nos3* expression via elevated expression of HO-1 (encoded by *Hmox1*) and via Ang-(1-7)/Mas receptor mediated mechanisms as well as elevated H_2_S production due to increased gene expression of all three H_2_S producing enzymes (*Mpts*, *Cth*, *Cbs*) lead to a reduction in oxidative load. The results also suggest that MLN-4760 does not induce inflammation, as the expressions of the inflammatory genes determined in this study (*Il1b*, *Tnf*, *Nos2*, *Ptgs1*, *Ptgs2*) and the anti-inflammatory gene *Pparg* were unchanged (dash lines).

Clinical data suggest that ~40% of patients with COVID-19 develop neurological symptoms associated with neuroinflammation and neuronal damage [53]. Xia et al. showed enormous oxidative stress due to higher reactive oxygen species (ROS) in a murine neuroblastoma cell line treated with Ang II, which was reduced by overexpressing ACE2. On the other hand, ACE2 gene therapy reduced Ang II-mediated ROS formation in ACE2^−/y^ mice [54]. Another study found that i.c.v infusion of Ang-(1-7) markedly reduced the levels of malondialdehyde (marker of oxidative damage) and increased activity of antioxidant enzyme SOD in the brain of SHRs [55]. The antioxidant effects of the ACE2/ Ang-(1-7)/MasR pathway in the brain were confirmed by a study in which the ACE2 agonist xanthenone reduced oxidative damage and increased glutathione level (the main non-enzymatic antioxidant) in rat model of cerebral ischemia/reperfusion injury [56]. Based on the abovementioned studies, it can be assumed that inhibition of the ACE2/ Ang-(1-7)/MasR pathway may lead to pro-oxidative and pro-inflammatory processes that may be associated with damage of the central nervous system. Our results confirmed a part of the hypothesis related to pro-oxidative processes due to MLN-4760 treatment as we found increased CD level in the BS tissue (Figure 5). The increased lipid peroxidation in our study was associated by the increased expression of the *Gpx4* gene, whose product glutathione peroxidase 4 plays a key role in detoxication of lipid peroxides and prevention of ferroptosis [57]. In addition, our study revealed increased expressions of *Sod1*, *Sod2* and *Hmox1* genes encoding proteins which are important antioxidant enzymes [58,59]. Increased gene expression of antioxidants might represent compensatory mechanisms suppressing oxidative damage caused byMLN-4760 treatment. In addition, increased oxidative damage due to oxidative stress correlated with increased gene expression of the nuclear transcription factor NRF2 (encoded by *Nfe2l2* gene), which upregulates the gene expression of the aforementioned antioxidant enzymes [60]. Our findings are supported by positive correlations between *Nfe2l2* gene expression and expressions of *Sod1*, *Sod2*, *Gpx4* and *Hmox1* genes encoding antioxidant enzymes. Thus, results suggest that MLN-4760 might act as pro-oxidants that activate NRF2 production followed by activation of antioxidant defence system as suggested in the Figure 5.

The analysis of gene expressions in our study did not confirm the pro-inflammatory effects due to MLN-4760 treatment, as we did not find the either increased expression of *Il1b* and *Tnf* genes encoding cytokines or *Ptgs1* and *Ptgs2* genes encoding prostaglandin-producing enzymes in the BS, all of which participate in the inflammatory reaction [61,62]. Expression of *Pparg*, which is known to act as anti-inflammatory factor, was also unaltered in the MLN-4760-treated rats [63]. The absence of pro-inflammatory effects of the MLN-4760 in the brain was also in agreement with unchanged gene expression of inducible NOS (*Nos2*) which is involved in inflammatory processes, while the elevated production of NO by endothelial NOS (encoded by *Nos3* gene) has anti-inflammatory effects [64,65,66,67]. In addition, increased NOS activity correlated positively with elevated *Nos3* expression and CD level but not with *Ace2* or *Nos2* expression. The increased NO production, positively correlated with endothelial NOS and oxidative damage, may represent compensatory mechanism in an attempt to suppress oxidative stress induced by MLN-4760 in the BS as studies indicate that NO inhibits chain lipid peroxidation and oxidative damage to the cell membranes [68]. The elevated NO production by eNOS activation can result from elevated *Nos3* expression via both of HO-1 and Mas receptor-mediated pathways [69,70].

We also investigated MLN-4760-induced changes in H_2_S release as recent studies pointed out that reduced H_2_S availability is a characteristic feature of COVID-19 while patients who had persistently elevated plasma H_2_S levels had a lower risk of adverse COVID-19 outcomes [71,72]. We found an increase in plasma H_2_S levels after MLN-4760 treatment. A similar increase in H_2_S formation after inhibition of ACE2 was found in the cardiac tissue [34]. In our study, H_2_S levels were determined in plasma, which reflect systemic levels of H_2_S. Although this does not reflect H_2_S production in the BS, we found positive correlations between the plasma level of H_2_S and expression of *Mpst*, *Cth* and *Cbs* genes encoding H_2_S-producing enzymes as well as increased gene expression of these genes after MLN-4760 treatment. Increased H_2_S formation may also represent another mechanism participating in suppression of oxidative stress induced by MLN-4760. Several studies have described the anti-inflammatory and antioxidant effects of H_2_S in the brain [73,74]. The antioxidant effect of H_2_S could also be mediated by increased expression of NRF2 protein [75]. The direct mechanism by which MLN-4760 stimulated the gene expression of H_2_S-producing enzymes and/or H_2_S production in the BS is unclear. However, we found positive correlations between *Nfe2l2* gene expression and expressions of *Mpst*, *Cth* and *Cbs* genes, suggesting the regulatory role of NRF2 in expression of these genes. Such a mechanism of NRF2-regulated gene expression of H_2_S-producing enzymes was described previously in in vitro [76,77] studies or in mice subjected to ischemia-induced chronic heart failure [78].

## 5. Limitations

Limitation of this study is that we did not measure diastolic blood pressure and protein expressions of the above-mentioned enzymes to confirm their elevated translation. We also did not investigate effects of MLN-4760 in normotensive rats which would reveal whether our findings are generally valid, or whether the activation of NRF2-dependent compensatory mechanisms is present only in SHRs.

## 6. Conclusions

In conclusion, our study is the first to show that chronic low-dose s.c. infusion of specific ACE2 inhibitor MLN-4760 induced pro-oxidative but not pro-inflammatory effects in the BS of SHRs. Pro-oxidative effect of MLN-4760 was accompanied by activation of compensatory mechanisms associated with elevated systemic H_2_S levels and stimulated NO production in the BS. In addition, MLN-4760 elevated gene expression of antioxidant enzymes and genes involved in H_2_S production in the BS, which all correlated positively with *Nfe2l2* expression, suggesting an important regulatory role of NRF2 in H_2_S release. Our results also showed that MLN-4760 treatment did not lead to the exacerbation of pre-existing hypertension and behavioural disturbances in SHRs. However, further research is needed to determine whether administration of a low dose of MLN-4760 can be used in the treatment of COVID-19 with the risk of inducing oxidative stress followed by activation of NRF2-mediated antioxidant mechanisms in the BS.

## Figures and Tables

**Table 1:** Used primer pairs in the qPCR.

Gene	Forward Primer	Reverse Primer	Tm (°C)	Amplicon Size (bp)
*Nos1* (NM_052799.1)	GCA GAG GCC GTC AAG TTC T	GAG AAT GGT CGC CTT GAC CC	60	72
*Nos2* (NM_012611.3)	AAA CGC TAC ACT TCC AAC GC	TGC TGA GAG CTT TGT TGA GGT C	59	91
*Nos3* (NM_021838.2)	GAT CCC CCG GAG AAT GGA GA	TCG GAT TTT GTA ACT CTT GTG CT	60	105
*Nfe2l2* (NM_031789.2)	TGC CAT TAG TCA GTC GCT CTC	ACC GTG CCT TCA GTG TGC	60	102
*Pparg* (NM_013124.3)	CTC ACA ATG CCA TCA GG TTT GG	GCT GGT CGA TAT CAC TGG AGA T	59	84
*Sod1* (NM_017050.1)	CTG AAG GCG AGC ATG GGT TC	TCC AAC ATG CCT CTC TTC ATC C	60	131
*Sod2* (NM_017051.2)	GCT GGC CAA GGG AGA TGT TAC	TGCTGTGATTGATATGGCCCC	60	83
*Gpx4* (NM_017165.4)	TAA GTA CAG GGG TTG CGT GTG	CAA GGG AAG GCC AGG ATT CG	60	135
*Hmox1* (NM_017165.4)	AGA AGA GGC TAA GAC CGC CT	TCT GGT CTT TGT GTT CCT CTG TC	60	86
*Mpst* (NM_138843.2)	GGC ATC GAA CCT GGA CAC AT	GGC GTT GGA TCT CCT CTG G	60	100
*Cth* (NM_017074.2)	GTA TGG AGG CAC CAA CAG GTA	GGT TGG GTT TGT GGG TGT TTC	60	151
*Cbs* (NM_012522.2)	ATG GTG ACT CTC GGG AAC ATG	AGG TGG ATC GGC TTG AAC TG	59	104
*Tnf* (NM_012675.3)	CGT CAG CCG ATT TGC CAT TTC	TGG GCT CAT ACC AGG GCT T	60	116
*Il1b* (NM_031512.2)	CAC CTC TCA AGC AGA GCA CAG	GGG TTC CAT GGT GAA GTC AAC	60	79
*Ptgs1* (NM_017043.4)	AGC ACA TTC GGT GGT GAT GT	GGG TAA TCT GGC ACA CGG AA	60	116
*Ptgs2* (NM_017232.3)	CTA CCA TCT GGC TTC GGG AG	TGG AAC AGT CGC TCG TCA TC	60	85
*Rpl10a* (NM_031065.1)	TCC ACC TGG CTG TCA ACT TC	GGC AGC AAC GAG GTT TAT TGG	60	134
*Ace2* (NM_001012006.2)	TCA GAG CTG GGA TGC AGA AA	GGC TCA GTC AGC ATG GAG TTT	60	111
*Mas1* (NM_012757.2)	TTC ATA GCC ATC CTC AGC TTC TTG	GTT CTT CCG TAT CTT CAC CAC CAA	60	84

Abbreviations: Tm, melting temperature; bp, base pair of DNA.

**Table 2:** Hemodynamic parameters of experimental animals.

Parameter	Cont Group *n* = 11	MLN Group *n* = 11
Basal systolic BP (mmHg)	146 ± 3	148 ± 4
End systolic BP (mmHg)	158 ± 8	157 ± 8
Basal HR (bpm)	531 ± 18	559 ± 13
End HR (bpm)	514 ± 25	520 ± 24

Values represent the mean ± SEM. Data were analysed by repeated measures ANOVA and followed with Bonferroni’s post hoc test. Abbreviations: BP, blood pressure; HR, heart rate; bpm, beats per minute.

## Data Availability

The data presented in this study are contained within the article and available on request.
